# Artificial Intelligence in Emergency Medicine: Viewpoint of Current Applications and Foreseeable Opportunities and Challenges

**DOI:** 10.2196/40031

**Published:** 2023-05-23

**Authors:** Gabrielle Chenais, Emmanuel Lagarde, Cédric Gil-Jardiné

**Affiliations:** 1 Bordeaux Population Health Center INSERM U1219 Bordeaux France; 2 Bordeaux University Hospital Bordeaux France

**Keywords:** viewpoint, ethics, artificial intelligence, emergency medicine, perspectives, mobile phone

## Abstract

Emergency medicine and its services have reached a breaking point during the COVID-19 pandemic. This pandemic has highlighted the failures of a system that needs to be reconsidered, and novel approaches need to be considered. Artificial intelligence (AI) has matured to the point where it is poised to fundamentally transform health care, and applications within the emergency field are particularly promising. In this viewpoint, we first attempt to depict the landscape of AI-based applications currently in use in the daily emergency field. We review the existing AI systems; their algorithms; and their derivation, validation, and impact studies. We also propose future directions and perspectives. Second, we examine the ethics and risk specificities of the use of AI in the emergency field.

## Introduction

### Emergency Services Crowding Effects

Emergency departments (EDs) and related services such as intensive care units and emergency medical dispatch (EMD) have recently been in the spotlight owing to the COVID-19 pandemic. The fragility of the emergency system has been exposed by overcrowded services, extensive waiting times, and exhausted staff struggling to respond to exceptional situations. Even during times of regular activity, the national efforts to improve waiting times and optimize the health care pathway for patients have underscored the necessity of reconsidering the emergency system. Indeed, the number of ED visits worldwide has increased faster than the rate of population growth in the past decades [1-3]. The identified causes of increasing ED attendance include nonurgent visits, frequent visitors, extended boarding times, staff shortages, and repeated reductions of downstream beds [4]. The negative effects of ED crowding include impact on several patient-oriented outcomes such as mortality [5-7], complication rates [1], walkouts [8], time to treatment [1,9], satisfaction [10], and length of stay [11]. Furthermore, ED crowding has been identified as a major stress factor for health care professionals, leading to burnout [12] and medical errors [13]. So far, solutions and efforts have mainly focused on improving patient workflow within the ED; however, a more comprehensive approach appears more effective [14]. Solutions provided by artificial intelligence (AI) could be one of the building blocks of a system-wide improvement for emergency medicine and services.

### Novel Approaches for Reshaping Emergency Medicine

**Figure 1:**
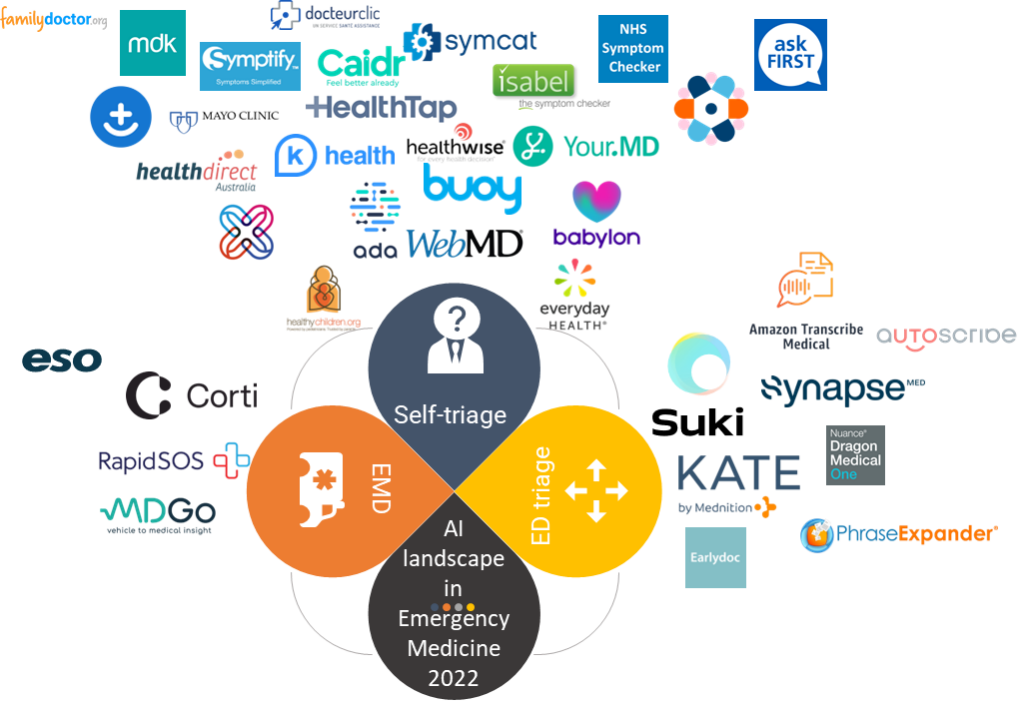
Artificial intelligence’s business landscape in emergency medicine in 2022. AI: artificial intelligence; ED: emergency department; EMD: emergency medical dispatch.

The field of emergency medicine has received considerable interest in the application of AI to health care owing to the unique nature of this medical practice. With challenges related to organization and coordination as well as the need for rapid and accurate decision-making for patients categorized as high acuity, novel approaches provided by AI are promising in emergency medicine and services. AI techniques have already been shown to be promising for improving diagnosis, imaging interpretation, triage, and medical decision-making within an ED setting [15]. However, most research on AI in emergency medicine is retrospective and has not led to applications beyond the proof of concept. Therefore, the potential for AI applications in routine clinical care settings is yet to be achieved. Critical appraisal of evidence supporting whether a clinical digital solution involving AI has an impact on patient outcomes should be mandatory [16]. Specifically, an independent evaluation by an objective independent entity (or authorized entities), both during development and use, should be performed. The independent evaluation would address verification, validation, and impact on patient outcomes and safety. To date, few system suppliers have challenged their products and services in terms of key health metrics [17]. However, some applications have already been deployed for prehospital, EMD, and ED (Figure 1). In this contribution, we attempt to depict the landscape of AI-based applications currently used in the daily emergency field. For each section, we will provide a context based on recent reviews, the AI applications’ algorithms or models used (if available), how they were validated, and whether the desired impact on patients’ outcomes was assessed. We also propose future directions and perspectives. Our second objective is to examine the legal and ethical specificities of AI use in the emergency field.

### Actual and Possible Applications of AI for Emergency Services

**Figure 2:**
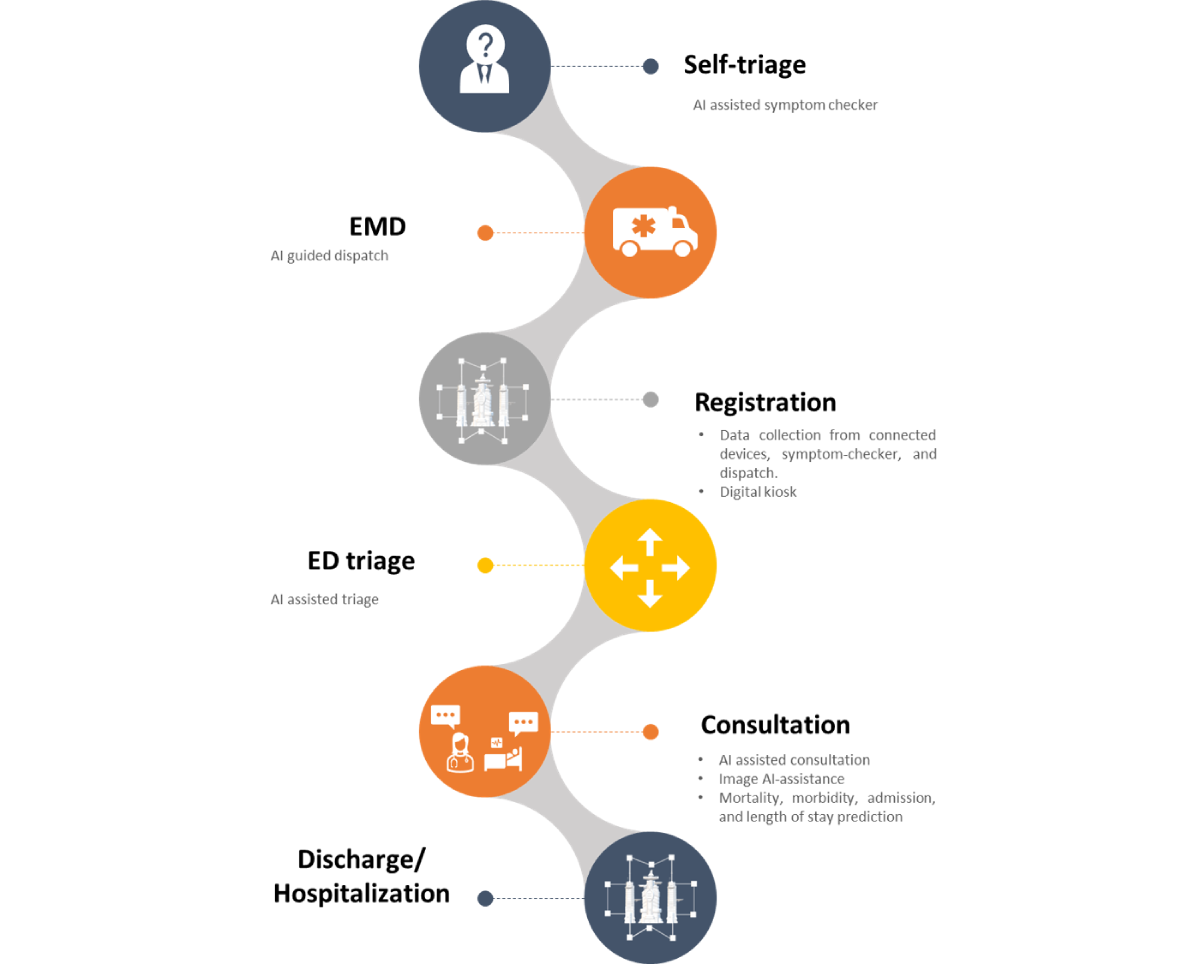
The emergency patient journey and where artificial intelligence is making or can make an impact. AI: artificial intelligence; ED: emergency department; EMD: emergency medical dispatch.

The journey of a patient who requires care in the ED includes several steps that can or could be impacted by AI (Figure 2). Before coming to an ED, several steps can be carried out such as checking symptoms on the internet and contacting the emergency call center or their general practitioner.

### Prehospital

#### Self-triage

The use of patient-facing clinical decision support systems (CDSSs) has continuously increased in recent years [18]. Tools assisting laypersons in their self-assessment of whether and where to seek urgent professional medical care and for what diagnoses based on the users’ input of symptoms and medical history are termed symptom checkers. To date, symptom checkers provided by free websites or mobile apps have proven to be inconsistent, supplying generally risk-averse advice and often recommending more urgent care than necessary [19,20]. Digital tools that impact care delivery and behaviors should undergo rigorous evaluation that enables evidence-based determination of their efficacy. However, evaluations of the effectiveness of self-sorting apps often provide limited evidence as they rely heavily on observational studies [21]. Schmieding et al [22] recently assessed the triage accuracy of 22 symptom checkers and showed that their performance did not improve between 2015 and 2020. For 2 cases of use, the triage performance decreased (advice on when emergency care is needed and when no health care is required for the moment). The apps sample of 2020 less frequently mistook self-care cases and nonemergency cases for emergencies; at the same time, it more often misclassified emergencies as nonemergencies [22]. Regarding the algorithms or models used by these proprietary websites or apps, information about their architecture, development, and validation is sparse. When the information is available, most symptom checkers and their decision support systems rely on probabilistic or graphical algorithms (Bayesian decision trees or Bayesian-directed graphs [23-28]). Some apps, such as Babylon Health [29], use a chatbot that presents the user with unique or multiple-choice questions for symptom assessment [30]. Although there is no clear explanation of the algorithm used by Babylon, the team has released open-sourced Neural Temporal Point Processed models [31], which are integrated into an encoder-decoder framework based on deep learning. This indicates that the app likely uses this type of model [32]. To ensure the safety of symptom checker users, transparency about the algorithms used should be maintained. Further research and development also seem necessary for improving these self-sorting tools. The use of deep learning models for these apps should be considered to attempt improving their limited efficacy (Textbox 1).

**Highlights of actual self-sorting and symptom checker apps and websites.**

- Multiple proprietary self-sorting apps
- Lack of validation studies
- Weak evidence for their efficacy
- Algorithms often undisclosed

#### Improving EMD

##### Overview

Prehospital emergency care and ambulance demands have substantially increased over the past decade [33-35]. EMD involves the receipt and management of demands for urgent medical assistance. It encompasses 2 main dimensions: call answering, where emergency medical calls are received and events are classified according to their priority (triaged), and coordinating, where the best available resources are dispatched to manage the event.

##### EMD Data Entry

Emergency medical dispatchers at EMD centers play a pivotal role in coordinating prehospital care. The interaction between the dispatcher and patient results in documentation that can be guided (structured form), semiguided (semistructured), or free (unstructured). Although effective in narrow and predictable domains, structured data entry can be quite slow when events are wide ranging and heterogeneous. To address this issue, the already-in-use Corti [36] system assists emergency dispatchers by analyzing the caller’s speech and description. This system provides advice on which questions to ask next, indicating when a patient may have a particular presentation, such as myocardial infarction or stroke. It also helps in data extraction, where the system can extract and pull information on the caller’s address and location to reduce the time needed to complete the call and dispatch emergency medical services. The framework of Corti contains 2 models: an automatic speech recognition (ASR) model that transcribes speech to text and an out of hospital cardiac arrest (OHCA) detection model that predicts OHCA events from transcribed speech in real time. The ASR is a deep neural network using a model based on Connectionist Temporal Classification [37]. This end-to-end (E2E) deep learning framework is based on a recurrent neural network, and the network outputs are transformed into a conditional probability distribution over label sequences (letters, words, or sentences of the caller). The network can then be used as a classifier by selecting the most probable label for a given input sequence [38]. For each second of raw audio, the classifier predicts whether there is an OHCA based on the accumulated audio sequence [36]. The efficacy of the AI-guided system provided by Corti was assessed for OHCA by Byrsell et al [36], and it was shown that the E2E model recognized OHCA faster than dispatchers. Despite the promising results for OHCA, the study assessing the system was retrospective, and other critical conditions were not tested.

Semistructured or free-structured text observations are the most frequently used input format for EMD, according to Miller et al [39]. If dispatchers require this format to be continued in the future, solutions to facilitate, speed up, and optimize this type of input should be considered. Computed free text involves natural language processing (NLP), and a recent breakthrough revolutionized this area in 2018 when the Transformer architecture was introduced by Vaswani et al [40] in “Attention is all you need.” The Transformer aims to solve sequence-to-sequence tasks while easily handling long-range dependencies (problems for which the desired output depends on inputs presented at times far in the past). It relies entirely on self-attention to compute its input and output representations without using sequence-aligned recurrent neural networks or convolutions. The Transformer architecture has evolved, and some models such as the Bidirectional Encoder Representations from Transformers [41] and the Generative Pretrained Transformer 2 [42] have achieved unprecedented performances on various NLP tasks such as classification, question answering, named entity-recognition, relation-extraction, or sentence-similarity tasks [43,44]. A major efficient feature of Transformers that dispatchers could benefit from is text generation through autocompletion [45,46]. By proposing a text complement fitting the string of characters that the dispatcher would have started to type, the autocomplete would allow to speed up the typing process and thus save time for the dispatcher. The autocomplete would also limit typing errors by entering the characters that remain to be typed without human intervention. Finally, the autocomplete would avoid the dispatcher having to correct their typing errors if necessary.

##### EMD Call Waiting Time

EMD calls can increase drastically under exceptional circumstances such as mass shooting, wildfires, or when it is recommended to call the center before seeking care (eg, COVID-19) [47,48]. To reduce the waiting time before reaching a dispatcher for very acute patients in ordinary and exceptional situations, some solutions such as prioritized queue with the help of an ASR model and a classifier are starting to be considered and designed [49]. To the best of our knowledge, such solutions have not been tested or even developed yet.

##### EMD Triage and Ambulance Dispatch

A large proportion of prehospital deaths when emergency medical services are involved are preventable, with 4.9% to 11.3% potentially preventable deaths and 25.8% to 42.7% definitely preventable deaths, as shown by Pfeifer et al [50]. The most frequent reasons evoked in this systematic review were delayed treatment of patients with trauma (27%-58%), management errors (40%-60%), and treatment errors (50%-76.6%) [50]. Treatment delays and caller management are often the result of dispatch algorithms that provide triage of patients categorized as high acuity for critical care and patients categorized as low acuity for diversion or nonurgent transport. Most of the current dispatch algorithms are rule based or encompass a human review of rule-based algorithms [39]. To date, 2 retrospective studies have shown that statistical machine learning and deep learning can improve or outperform rule-based algorithms [51,52]. Further validation and impact studies are needed to improve the current dysfunctional EMD triage, and AI should be considered for enhancing the dispatch algorithms. Start-up companies are making proposals to help reduce response times and ensure data transmission from connected devices before or during calls. For example, the RapidSOS system is an emergency response data platform that securely links data from connected devices and sensors directly to first responders during emergencies. Another promising system provided by the Israeli start-up MDGo is the use of advanced AI technology to help dispatchers know if a car accident requires an ambulance. When a car crash occurs, the system creates a medical report in real time with data regarding the forces applied on the passenger (eg, duration, moment, and vector). These data are sent automatically to the Israeli emergency medical services.

### Improving EDs

#### ED Registration and Redirection

Whether generated from a symptom checker with a self-triage step, from a call to an EMD center, or a connected device, all collected data concerning patients could benefit EDs. Linking emergency medical services to ED data allows a continuum of care assessment and improvement in patient outcomes [53]. Concerns regarding interoperability, security, accurate patient match algorithms, and the reliability of wireless networks as potential barriers to adoption were identified in a review conducted by Martin et al [54]. Several studies have demonstrated the feasibility of various statistical models for electronic health record (EHR) linking with EMD systems [54]. For example, Redfield et al [55] used logistic regression to link Boston’s EMD electronic patient care reports with their hospital EHR and achieved an unprecedented success rate of linkage without manual review (99.4% sensitivity). The next few years will likely reveal an expansion in the use of these techniques in new ways. For patients arriving at the ED by their own means, an initial medical screening could be performed by asking a small number of questions using a smartphone or a digital kiosk set up at the ED entrance. To date, all trials entailing the redirection of patients categorized as low acuity within EDs involved human intervention and were unsuccessful or discontinued owing to adverse public relations incidents [14,56]. In a fully digitalized world, the acceptance of such solutions accompanied by awareness campaigns should be more substantial.

#### ED Triage

**Figure 3:**
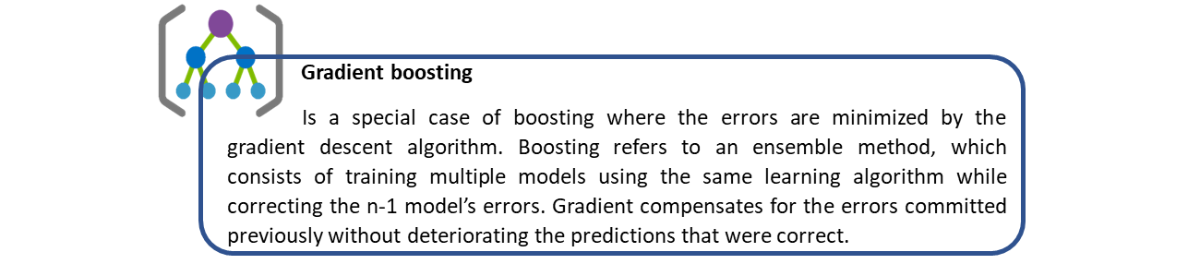
Gradient boosting explanation.

The check-in desk at the entrance of the ED is the first point of contact for a patient requiring emergency care where administrative agents open a specific section of the EHR. The patient then becomes a future occupant of the ED room or cubicle after being assessed by the triage nurse. Triage is a sorting process in which the “triage nurse” is required to quickly assess a large number of patients to decide the urgency of their condition and the location in the ED in which they will be evaluated and treated. Triage includes the attribution of a triage score to each patient, and several scales have been developed worldwide, with no evidence of superiority for one of them [57,58]. Even with the adoption of 5-level triage scales, the assessment still relies heavily on the subjective judgment of the triage nurse, which is subject to significant variation [59]. Furthermore, Hinson et al [60], in their systematic review, found several studies reporting low sensitivity (<80%) in identifying patients who had critical illness outcomes or died during the hospitalization. To address the lack of accuracy in the triage process, several AI-based solutions have been tested, and the authors found that there was an improvement in the health care professionals’ decision-making, thereby leading to better clinical management and patient outcomes [61,62]. However, these solutions were not dedicated to triage but outcomes such as hospital admissions, mortality, or ED length of stay. An example of a real-time AI application that is already used in 16 US hospitals is provided by KATE [63,64]. Unlike most proprietary software, a validation study has been published that showed that KATE’s accuracy using an extreme gradient boosting model [64] (Figure 3) was 27% (*P*<.001) higher than the average nurse accuracy. However, no impact study has yet been published.

Similar to dispatchers, the documentation workload of triage nurses can benefit from AI applications. Health care professionals currently spend up to 50% of their time documenting information in EHR [65-67]. The time spent performing documentation tasks induces both poor and inconsistent data, which may impact the quality of care [68,69]. Physicians prefer using free text over restrictive structured forms, but clinical notes often lack readability owing to an overload of acronyms and jargon [70,71], which leads to noisy, ambiguous, and incomplete data.

A first improvement lever could be autocompletion, which combines automatic annotation with labels of clinical concepts. Greenbaum et al [72] and Gopinath et al [46] set up the foundations of such technologies. The Massachusetts Institute of Technology clinical machine learning group, led by Gopinath et al [46], developed a tool called Medknowts that aims to autocomplete clinical terms in the EHR while note-taking. This tool was assessed in a real ED environment and showed a 67% reduction in the keystroke burden of clinical concepts [46]. The model used is fully disclosed and is based on a shallow dual branch neural network for a minimal latency (time taken to process 1 unit of data) of approximately 0.2 milliseconds. In addition, MedKnowts allows the retrieval and display of context-specific information from a patient’s EHR while unifying the documentation and search process [73]. However, the language aimed to be autocompleted with these systems is strictly medical and does not reflect the reality of clinical notes containing both nonmedical and medical concepts. Using new NLP deep learning models such as Transformers, as mentioned previously, can help handle the complexity of these type of data. Transformers have reached a state-of-the-art status for ASR by reducing the word error rate to <5 (the lower the better) on several libraries and languages [74]. Nonetheless, some challenges remain to be addressed such as latency, streaming, and adaptation capabilities for implementing E2E models. The growing progression in the technological capabilities of hospitals (servers and graphics cards) will allow for real-time efficiency without affecting the workflow. Another solution is to retrieve relevant information from real-time dialogues between health care professionals and patients. Ideally, the system would write down information in free-text form but would also extract entities such as symptoms or medications and predict scores, risk factors, and diagnosis. Vocal AI assistants such as Suki [75] and Dragon Medical One [76] are already available for health care practitioners, claiming a documentation time reduction of 72%. So far, no peer-reviewed derivation or validation studies have been found to support the legitimacy of these solutions’ commercial claims.

### The Digital Hospital Concept

**Figure 4:**
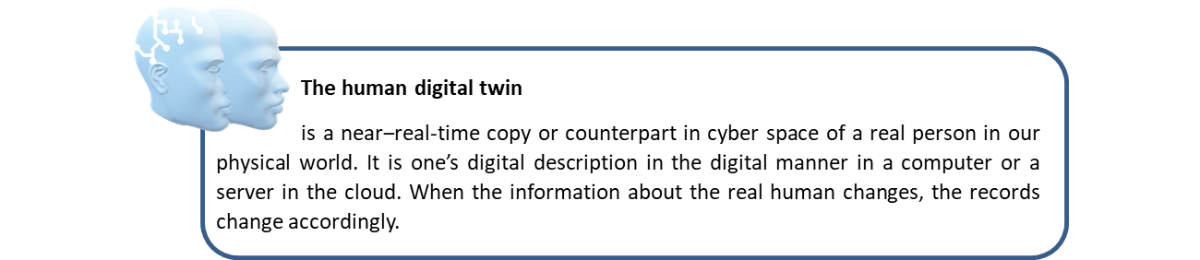
The human digital twin.

A digital hospital concept in the image of the digital twin [77] (Figure 4) would allow real-time bed availability. The admission and discharge data, currently collected by the admissions departments, could be transferred to the digital hospital, and the estimation of the projected bed availability rate could be made available in each department. Traditional models estimating length of stay are mostly statistical [78] or based on machine learning [79] using the previous length of stay as input. The digital hospital model would be based on the same foundation and would also be adjusted regularly owing to a trend toward shorter lengths of stay and a shift to ambulatory medicine. The model would also be able to adjust to external data such as environmental and epidemiological factors (eg, epidemics) in real time. Thus, if visibility on downstream beds is guaranteed, not only can waiting time in the ED be reduced when hospitalization is needed, but transfers to downstream services can also be facilitated in the event of congestion. Creating a network of all digital hospitals at the regional or state level could ensure the availability and visibility of beds and facilitate transfers between health care facilities. On a comprehensive scale, these data can provide real-time visibility of foreseeable ED arrivals and allow resources to be adapted accordingly.

### Improving the Patient’s Waiting Time Experience

Patient experience or satisfaction with ED care is a growing area of research, and the literature has demonstrated a correlation between high overall patient experience and improved patient outcomes, cost-effectiveness, and other health care system goals [80-82]. Several factors lead to better patient satisfaction in emergency medicine such as actual waiting times [83], perceived waiting times [84], staff-patient communication, and staff empathy and compassion [85].

Waiting time to care in ED is the cumulative result of the time from registration assessment and the time from assessment to the initiation of medical care. This waiting time is modulated by triage in EDs when dedicated triage staff are available. Inadequate staffing has been identified as a major throughput factor associated with longer waiting times [4]. Apart from alleviating documentation tasks and facilitating flow management in ED, AI cannot propose solutions when political decisions or executives regulate staff quotas. In contrast, perceived waiting time could benefit from innovation. Waiting without information provided about delays can be a tedious and frustrating experience among people seeking urgent care, and lack of information magnifies patients’ sense of uncertainty and increases their psychological distress sometimes, leading to violent behaviors [86,87]. Transparency is a major determinant of patient satisfaction related to waiting time [12,52]*.* Patients provided with written or gamified ED processes tend to have a higher level of satisfaction [88,89]. Information about the estimated waiting time is provided by triage nurses or signboards at the admission desk in some hospitals. However, it has been shown that this information is not given for most patients [90]. Accurate waiting time for patients can be derived from the digital hospital with a dashboard of available places and beds. A screen indicating the waiting time in real time can be installed in the waiting room [91]. Additional information such as major events impacting the waiting time could be displayed on the screen (eg, a pileup on the highway), and mobilizing the patient’s empathy could reduce self-centered perception of care [92]. Patient-specific information on personalized waiting time estimates can also be provided via a mobile app. A positive environment can also improve a patient’s perception of waiting time [93]. Distracting activities such as the use of personal cell phones can be difficult for some patients in ED rooms. The benefits of virtual reality glasses have already been demonstrated in pain management [94] and in the reduction of preoperative anxiety [95]. Hence, virtual reality glasses can also be proposed for distraction and counseling.

### ED and EMD Data Processing Enhanced by AI for Public Health Surveillance

EDs and EMD centers generate a large volume of diverse health-related data. For public health surveillance aims, these data are most often used retrospectively and by sampling hospitals [96]. Some near–real-time surveillance systems use information extracted from EHR in addition to manual implementation provided by health care professionals [97]. These nonexhaustive procedures are time and resource consuming and are mostly based on voluntary work. Automatic signal extraction from EHR would allow real-time monitoring and ensure the responsiveness sought in any surveillance system [98,99]. The use of new state-of-the-art NLP models such as Transformers would bypass the difficulties in extracting fine-grained and standardized data from the most frequently used entries (free text) in ED and EMD.

Furthermore, with the appropriate network infrastructure, data should be collected and analyzed in real time, enabling early, accurate, and reliable signals of health anomalies and disease outbreaks. In addition, AI provides an opportunity to use various new or underexploited data sources for public health surveillance purposes, particularly those not originally or intentionally designed to answer epidemiological questions. A large amount of nontraditional data are self-generated by the public through their ubiquitous use of smart devices and social media. Public health has the potential to use real-time longitudinal data collected for health surveillance [100].

### Ethical and Legal Challenges Posed by the Implementation of AI in Emergency Medicine

#### Overview

**Figure 5:**
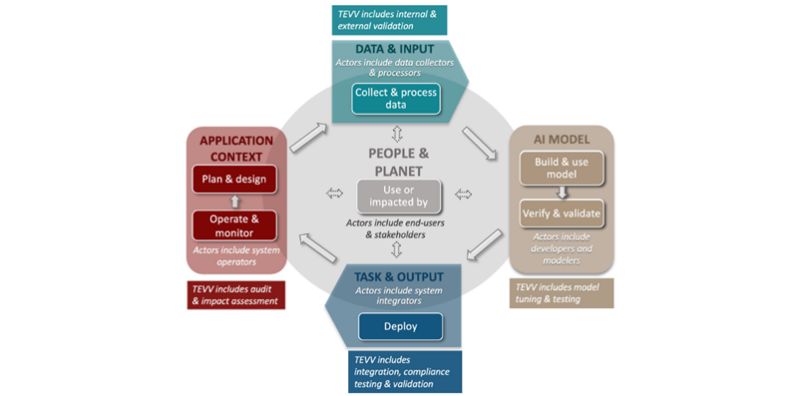
Life cycle and key dimensions of an artificial intelligence (AI) system. Extracted from National Institute of Standards and Technology [101]. TEVV: test, evaluation, verification, and validation.

Despite the potential of AI to improve emergency clinical care, numerous ethical and legal challenges prevail. An ethical principle is a statement of a duty or a responsibility, and when applied to AI technologies for health, it covers their life cycle (Figure 5).

A trustworthy AI is safe and fair with managed biases, transparent and accountable, explainable and interpretable. AI protects human autonomy, and is privacy-enhanced [101,102]. A sense of common responsibility among all the actors involved in an AI life cycle should prevail, and health care providers have a special duty to adhere to these requirements because of patients’ dependence on their care, should AI systems be used to assist health care practitioners in clinical decision-making [103]. To lay the foundations for trustworthy AI in emergency medicine, the ethical considerations cannot be dissociated from the legal answers that are or will be provided.

#### Safety, Fairness, and Bias Management

AI systems “should not, under defined conditions, cause physical or psychological harm or lead to a state in which human life, health, property, or the environment is endangered” [104]. Identifying, mitigating, and minimizing risks and potential harms associated with AI applications, especially in emergency medicine, are essential steps toward the development of safe AI systems and their appropriate and responsible use [101].

**Figure 6:**
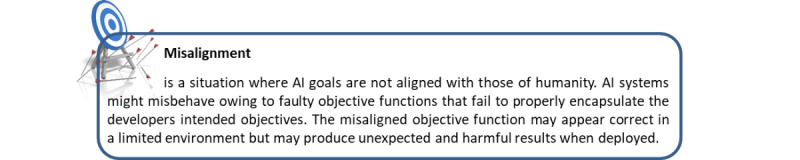
Misaligned goals in artificial intelligence (AI).

Addressing AI risks and bias prospectively and continuously throughout the AI life cycle aims at preventing misalignment (Figure 6) [105,106].

Current attempts to address the harmful effects of AI bias remain focused on computational factors. However, systemic, human, institutional, and societal factors are also important sources of AI bias and are currently overlooked. We hereby propose to initiate the discussion and lay the groundwork for managing the risks associated with the use of AI in emergency medicine by identifying the biases that can be anticipated.

#### Bias in Data and Design

Once end users (eg, health care professionals) start interacting with an AI system or application, any early design and development decisions that were poorly specified and based on narrow perspectives can be exposed, leaving the process vulnerable to additive statistical or human biases [107].

##### Data Set Bias Challenge

Several categories of biases are held by health data sets used for training AI.

First, the choice of the data set for either pretraining or training can produce a *sampling bias* leading to a distributional shift [108], which is a mismatch between the data or environment in which the system is trained and that used in operation. Would training an AI application on EHRs of a local ED in a given region or state with given protocols and EHR architecture lead to the same results in the neighboring state’s university hospital? When considering a physician-patient vocal assistant, how can language variety (regional or social dialects), linguistic variations (pronunciation, prosody, word choice, and grammar), and foreign speakers be considered?

Large-scale data sets are increasingly deployed for decision support applications, often in high-risk settings such as emergency medicine, and off-label uses result in *representation bias* harms. Low-represented populations or conditions should be carefully handled with rebalancing techniques such as data augmentation, oversampling, or weighting systems. Causal models and graphs can also be used to detect direct discrimination in the data [109,110].

*Aggregation bias* (or ecological fallacy) arises when false conclusions are drawn about individuals from observing the entire population. An example of this type of bias in an emergency setting would be patients calling or presenting themselves with heart failure. Symptoms of heart failure differ in complex ways across genders [111,112]. Therefore, a model that ignores individual differences will likely not be well suited for gender groups in the population. This is true despite an equal representation in the training data. Any general assumptions regarding subgroups within the population can result in aggregation bias [113].

The *Simpson paradox* should also be considered at the designing step. The Simpson paradox is a type of aggregation bias that arises in the analysis of heterogeneous data [114]. The paradox arises when an association observed in aggregated data disappears or reverses when the same data are disaggregated into their underlying subgroups. For example, if an AI-guided CDSS was to be built for naloxone administration, when testing the model, if the clinical presentation severity or opioid type is unequally distributed among groups, the Simpson paradox will likely contribute to different rates of naloxone administration [115].

*Modifiable areal unit problem* is a statistical bias in geospatial analysis that arises when modeling data at different levels of spatial aggregation [116]. This bias results in different trends learned when the data are aggregated at different spatial scales. For example, when designing an AI system for ambulance demand, only estimates based on minimal-resolution data should be relied upon, as ambulance demand using areal data is potentially misleading owing to the *modifiable areal unit problem* [117].

*Omitted variable bias* can also arise from variable selection for an emergency AI application. For example, when considering a triage application in which care protocols and treatment guidelines vary based on the patient’s insurance status, omitting this variable could lead to errors in the triage score. However, considering this variable for better accuracy will lead to unfairness, which is already present in a real-world setting.

High-quality input data are essential for constructing realistic AI systems. *Missing data bias* is common in EHR data input quality management, and its gestion should be considered during the design step [118]. Several authors suggest that explicitly representing the presence or absence of data in the underlying logic of a CDSS can improve prediction performance [119].

Owing to the specificity of ED activities, data entry also comes with several biases such as *recall bias* (as health care practitioners often enter data several minutes or hours after the emergency has occurred) or *confirmation bias* (as health care practitioners often rely on heuristic-based decisions [120]). It has recently been shown that serious games can improve physicians’ heuristic judgment by providing them with a simulated experience. Additional experiments could lead to better data capture for less biased data sets [121].

*Human biases*, whether conditioned socially or cognitive, may influence data selection, preprocessing, annotation (attributing labels to an unlabeled data set), and analysis process. Annotator biases could lead to biases in the training or test data set. Hence, proper training on the annotation task, sufficient incentives, facilitating background and expertise diversity among annotators (eg, nurses, physicians, researchers, and students), and the inclusion of a follow-up procedure with agreement evaluation could help in reducing these *label biases* [122].

*Systemic institutional biases* are also expected in the health data sets used to model the underlying AI applications. The issue of “flattening” the societal and behavioral factors within the data sets themselves is problematic but often overlooked [123]. If these biases are left unattended, AI applications are likely to reproduce human bias such as triage errors for women, older adults, and minor ethnicities [124,125].

##### Bias in AI Model Choice and Validation

The choice of models and their training process is a crucial step in the AI life cycle, and multiple biases can result from this. Most AI applications presented in the *Actual and Possible Applications of AI for Emergency Services* section are based on NLP, and concerns regarding the biases introduced by the growing use of large language models (ie, Bidirectional Encoder Representations from Transformers, Generative Pretrained Transformer 2, and XLNET) are relevant [126].

##### Semantic Biases

Embeddings are the most common text inputs represented in NLP systems, and they have been shown to detect racial and gender biases in training data [127]. As large language models are pretrained on almost the entire text corpus available from the internet, they are prone to the same societal biases as those that prevail on the internet. Semantic biases hold not only for word embeddings but also for contextual representations. Debiasing sentence representation is at the heart of the efforts of some research teams. However, the impact and applicability of debiased embeddings are unclear for a wide range of downstream tasks [128].

##### Algorithmic Effect

The algorithmic complexity can vary greatly from one AI model to another. The number of parameters that mathematically encode the training data can range from 1 to 1 trillion. Simple models with fewer parameters are often used because they tend to be cheaper to build, have better latency and better generalizability, are more explainable and transparent, and are easier to implement. However, these models can exacerbate statistical biases because restrictive assumptions about the training data often do not hold with nuanced demographic data. Complex models are often used for nonlinear and multimodal data such as text and images. These models can capture latent systemic biases in ways that are difficult to recognize and predict. Expert systems, another AI paradigm, can encode cognitive and perceptual biases in the accumulated knowledge of practitioners from which the system is designed to draw.

##### The Objective Function Bias

The choice of the model’s objective function, upon which the model’s definition of accuracy is based, can reflect bias. In an emergency context, decisions must often be taken rapidly, meaning that AI should not increase the time required to reach a decision that would divert the patient to appropriate care. Not taking the vital and time context into consideration during model selection could harm patients. In addition to task-specific metrics, streaming and adaptation must be considered.

##### Validation Bias

Performing tests on an AI system involved in health care under optimal conditions is challenging. Rigorous simulation and in-domain testing of time-specific windows or given locations should be performed before generalization. Randomized controlled trials and prospective studies in compliance with guidelines specific to AI interventions such as CONSORT-AI (Consolidated Standards of Reporting Trials–AI) [129] or SPIRIT-AI (Standard Protocol Items: Recommendations for Interventional Trials–AI) [130] should be conducted to ensure the transparency and validation of the application. The CONSORT-AI extension recommends that investigators provide clear descriptions of the AI intervention, including instructions and skills required for use, the setting in which the AI intervention is integrated, the handling of inputs and outputs of the AI intervention, the human-AI interaction, and the analysis of error cases.

#### Bias in Deployment

##### Inclusiveness Bias

AI should encourage equitable use in emergency and primary care independent of age, gender, ethnicity, income, language spoken, or ability to comprehend. When considering a smartphone app or a digital lock at the entrance of an ED, different languages should be proposed. Accessibility devices for disabilities (visual, hearing, moving, and reading impairments) should also be made available. Access to these technologies is particularly challenging for older adults, and alternative solutions should be proposed for this population.

##### Automation Complacency

Health care practitioners may have a propensity to trust suggestions from AI decision support systems, which summarize large numbers of inputs into automated real-time predictions, while inadvertently discounting relevant information from nonautomated systems. Some information about the visual, behavioral, and intuitive analysis of a patient does not necessarily lead to rigorous documentation in EHR, yet this information contributes to clinical decision-making. Moreover, can this type of information can be captured by an AI model? Fully relying on a triage score prediction provided by an AI application without the necessary hindsight toward the added value of one’s experience, common sense, and observation skills could lead to inaccurate resource allocation or priority levels for patients during triage.

##### Selective Adherence

In contrast, health care practitioners can selectively adopt the AI advice when it matches their preexisting beliefs and stereotypes, leading to biases in the overall performance of the system.

##### Monitoring

Continuous measurement and monitoring of an algorithm’s performance is necessary to assess whether it has a detrimental impact on patients or groups of patients. Tests and evaluations should cover the potential differential performance of the model according to age, gender, and relevant characteristics. As health care facilities benefit from quality and safety certification by public health and governmental agencies, AI technologies in health care should be audited periodically and externally. The report of these evaluations should be made public and intelligible to ensure transparency. In addition, assessing algorithm errors or deviations from human decisions can lead to reinforcement learning and an improvement in the model. Safe AI refers to the ability to modify misaligned systems. For this purpose, adversarial training procedures should be developed both as part of the training phase and the implementation.

#### Fairness and Inclusiveness

*Fairness* in AI includes concerns for equality and equity by addressing issues such as bias and discrimination. Fairness standards can be complex and difficult to define in emergency medicine because of disparities across health care systems (eg, in the United States, where hospital care protocols and treatment guidelines vary depending on the patient’s insurance status), policies, and geographic areas.

*Inclusiveness* requires that AI used in health care be tailored to support the broadest possible appropriate and equitable use and access, regardless of age, gender, income, ability, ethnicity, language spoken, or ability to comprehend. AI should be developed, deployed, and monitored by people from diverse disciplines, expertise, backgrounds, and cultures. AI technology should be designed and evaluated by those required to use the system including patients (who are themselves diverse).

#### Transparency, Accountability, and Liability

In the interest of patient safety and trust, a certain amount of *transparency* must be ensured. Transparency reflects the extent to which information about an AI system or application is available to individuals. Its scope ranges from design decisions to training data, the structure of the model, its intended use case, and how and when deployment or end-user decisions were made and by whom. Transparency and participation can be increased by the use of open-source software for the underlying design of an AI technology or by making the source code of the software publicly available (eg, Babylon Health). However, there may be some legitime issues related to intellectual property protection [131].

The use of AI technologies in health care requires the assignment of responsibility within complex systems in which responsibility is distributed among different actors. When medical decisions made by AI technologies harm individuals, the responsibility and *accountability* processes must clearly identify the relative roles of manufacturers and clinical users in that harm. This is an evolving challenge that remains unsolved in the laws of most countries [132]. Institutions have not only a legal responsibility but also a duty to take responsibility for the decisions made by the algorithms they use. To avoid the diffusion of liability, a seamless liability model (“collective responsibility”), in which all stakeholders involved in the development and deployment of an AI technology are held accountable, can encourage all actors to act responsibly and minimize harm. Another proposition made by Maliha et al [133] is the creation of a compensation program that does not consider liability but instead assesses fees on stakeholders.

Health care practitioners and health systems may be liable for malpractice or negligence. Imagine a dispatcher fully relying on an AI application that did not correctly classify the patient as high risk of having an OHCA, inducing delay in assistance and eventually death. To what extent would the dispatcher be liable for malpractice? So far, tort law protects health practitioners from liability as long as they follow the standards of care, regardless of its effectiveness in a particular case. AI involvement in emergency medicine has induced a previously unregulated paradigm shift. Possible legal outcomes depend on whether the AI application’s recommendation follows the standard of care and on the AI accuracy, practitioner action, and patient outcome, as proposed by Price et al [134] (Table 1).

Clinical malpractice, whether involving AI or not, leading to injury often induces compensation, as mentioned in Table 1. ED physicians already have higher rates of malpractice insurance owing to the higher risk of lawsuits. Does the malpractice insurer encompass the use of AI in high-risk fields such as emergency medicine? If so, how do we ensure that health care professionals receive the necessary insurance coverage? How can health care professionals be defended in court when they are threatened by claims involving AI? These questions remain to be answered by the legal community.

**Table 1:** Examples of potential legal outcomes related to artificial intelligence (AI) use in clinical practice [134].

AI recommendation and accuracy			Practitioner action		Patient outcome		Legal outcome (probable)
**Standard of care**							
	Correct	Follows		Good		No injury and no liability	
	Correct	Rejects		Bad		Injury and liability	
	Incorrect (standard of care is incorrect)	Follows		Bad		Injury but no liability	
	Incorrect (standard of care is incorrect)	Rejects		Good		No injury and no liability	
**Non–standard of care**							
	Correct (standard of care is incorrect)	Follows		Good		No injury and no liability	
	Correct (standard of care is incorrect)	Rejects		Bad		Injury but no liability	
	Incorrect	Follows		Bad		Injury and liability	
	Incorrect	Rejects		Good		No injury and no liability	

#### Explainability and Interpretability

*Explainability* refers to a representation of the mechanisms underlying the operation of an algorithm or model, whereas *interpretability* refers to the meaning of an AI system’s output. Laws and regulations such as the European General Data Protection Regulation (GDPR) state that automated (or guided) decision-making should come along with the logic involved, as well as the significance and the envisaged consequences of such processing for the data subject (Article 13{2}). When considering the possible application of emotion detection in voice during emergency calls to detect urgent conditions, the transparency and explainability of an AI solution is challenging. In emergency situations, the time requirements and explanation details collide. Thus, information regarding the outputs of an AI application should be meaningful and straightforward. Traditional machine learning models are mostly based on techniques that are inherently explainable. In contrast, deep learning models are considered as “black boxes” and have a higher computational cost (memory requirements and inference time). Explainable AI (XAI) is a recent field of research that attempts to provide solutions to confer trust in AI for practitioners [135]. XAI has additional features that enable better interpretability for end users. These features or explanations are provided for the model’s process as a whole (global) or for an individual prediction (local). This explanation emerges directly from the prediction process (self-explaining) versus processing post hoc [136]. Depending on the stakeholder’s expectations, the explanations and the way they are provided differ. There is a lack of consensus about which explanations can be used in different health care settings and how to measure them. Most studies have focused on subjective measurements, such as user satisfaction, goodness of explanation, acceptance, and trust in the system [137]. Further studies are required to evaluate the performance of XAI in health care settings.

#### Autonomy

##### For Emergency Health Care Providers

The adoption of AI in health care will lead to situations in which decision-making power can be, or is at least partially, transferred to machines. Protecting autonomy implies that humans remain in control of medical and health care system decisions. The opacity and “black-box” problem of an AI system [138] can make it difficult for health care professionals to ascertain how the system arrived at a decision and how an error may occur. How can health care providers be expected to remain in full control of their AI-assisted decisions when interpreting AI decisions is opaque even for developers? To what extent should health care providers inform patients that they do not fully interpret the recommendation provided by the AI system? AI systems should be designed to assist health care providers in making informed decisions. Moreover, to account for an AI application, ranking decisions and providing confidence score should be mandatory. For example, in the case of an emergency triage score, for each score proposed by an AI system, the predictions with highest accuracy should be given along with their associated probabilities.

##### For Patients

AI technology should not be used without the patient’s valid informed consent. Owing to the patient’s sometimes life-threatening condition, consent based on clear and intelligible information is not always feasible. Therefore, the responsibility for making an AI-assisted decision is shifted to health care professionals. Informed consent and its exceptions, without the use of AI, are equally regulated in the United States and Europe, with a tendency to not render practitioners liable for decisions taken in critical situations [139]. However, these statutory exceptions do not protect against litigation for malpractice and lack of informed consent [140]. Should health care practitioners use the AI-guided CDSS when obtaining informed consent is not possible? European Union has taken several steps to address the issue of liability when AI is involved in clinical decision-making. GDPR Article 13 (2): “[...] the controller shall, at the time when personal data are obtained, provide the data subject with the following further information necessary to ensure fair and transparent processing: (f) the existence of automated decision-making, including profiling, referred to in Article 22 (1) and (4) and, at least in those cases, meaningful information about the logic involved, as well as the significance and the envisaged consequences of such processing for the data subject.”

Under Article 22 (1) and (3), “The data subject (ie, the patient) shall have the right not to be subject to a decision based solely on automated processing, including profiling, which produces legal effects concerning him or her or similarly significantly affects him or her” unless the decision is “based on the data subject’s explicit consent.” However, the GDPR does not provide regulations for specific situations such as those mentioned in *Transparency, Accountability, and Liability* section, but the European Commission is currently working on a liability directive to address and regulate liability for AI use [141,142].

### Privacy

Privacy generally refers to norms and practices that help to preserve individual autonomy, identity, and dignity. Privacy-related values, such as anonymity, confidentiality, and control, should generally guide choices in the design, development, and deployment of AI systems. For example, the characteristics of AI and the novel risks associated with privacy protection are addressed in the European GDPR. Developing a compatible international framework to protect personal information would benefit stakeholders, and particularly patients, involved in AI for health care [143]. Clear information regarding the use of patient data for AI development purposes should be made available at any point of the emergency care trajectory. The right to erasure (right to be forgotten) as stated by GDPR Article 17 (“the data subject shall have the right to obtain from the controller the erasure of personal data concerning him or her without undue delay and the controller shall have the obligation to erase personal data without undue delay under given conditions”) should be made possible, although it is problematic for AI developers.

### Conclusions

AI has gained increasing attention owing to its potential advantages in health care and especially in emergency medicine for which several applications are currently used. Most ED and EMD AI applications are based on NLP and ASR because of the privileged documentation medium of free or semistructured text or the practitioner-patient interaction. There are limited studies on the types of models used and their validation methods. We noted a lack of evidence for symptom checkers with decreasing performance over time. Overall, AI-based applications in emergency medicine lack proper derivation, validation, or impact evaluations that are performed rigorously and independently.

Building a trustworthy, safe, and XAI requires a holistic approach that encompasses all sociotechnical aspects involved. Human factors such as participatory design and multistakeholder approaches are important for building such AI systems. Inclusiveness begins at the very beginning of the design step, with the inclusion of stakeholders (including end users) from diverse disciplines, expertise, backgrounds, and culture. All possible biases and risks should be identified and documented before any initiation, and they should be monitored continuously.

However, when emergency medicine is concerned with the development of AI applications, several principles mentioned above collide, and trade-offs must be determined. How can we determine the trade-off among interpretability and performance, time, and explainability? How can transparency be ensured when intellectual property is involved? How can liability be determined when AI harms?

AI should alleviate the high burden placed on health care professionals, but despite the ethical foundations laid, the actors gravitating around health care systems such as legislators, regulatory agencies, and insurers are not federated to ensure the safety of stakeholders.

## Abbreviations

AI: artificial intelligence
ASR: automatic speech recognition
CDSS: clinical decision support system
CONSORT-AI: Consolidated Standards of Reporting Trials–Artificial Intelligence
E2E: end-to-end
ED: emergency department
EHR: electronic health record
EMD: emergency medical dispatch
GDPR: General Data Protection Regulation
NLP: natural language processing
OHCA: out of hospital cardiac arrest
SPIRIT-AI: Standard Protocol Items: Recommendations for Interventional Trials–Artificial Intelligence
XAI: explainable artificial intelligence

## References

[1] Pines J, Pollack CV, Diercks DB, Chang AM, Shofer FS, Hollander JE (2009). The association between emergency department crowding and adverse cardiovascular outcomes in patients with chest pain. Acad Emerg Med.

[2] Richardson D, Kelly A, Kerr D (2009). Prevalence of access block in Australia 2004-2008. Emerg Med Australas.

[3] Hooker EA, Mallow PJ, Oglesby MM (2019). Characteristics and trends of emergency department visits in the United States (2010-2014). J Emerg Med.

[4] Hoot NR, Aronsky D (2008). Systematic review of emergency department crowding: causes, effects, and solutions. Ann Emerg Med.

[5] Chalfin DB, Trzeciak S, Likourezos A, Baumann BM, Dellinger RP (2007). Impact of delayed transfer of critically ill patients from the emergency department to the intensive care unit*. Critical Care Med.

[6] Richardson DB (2006). Increase in patient mortality at 10 days associated with emergency department overcrowding. Med J Aust.

[7] Sprivulis PC, Da Silva J, Jacobs IG, Jelinek GA, Frazer AR (2006). The association between hospital overcrowding and mortality among patients admitted via Western Australian emergency departments. Medical J Aus.

[8] Stock L, Bradley G, Lewis J, Baker D, Sipsey J, Stevens C (1994). Patients who leave emergency departments without being seen by a physician: magnitude of the problem in Los Angeles County. Ann Emerg Med.

[9] Pines J, Hollander J, Localio A, Metlay J (2006). The association between emergency department crowding and hospital performance on antibiotic timing for pneumonia and percutaneous intervention for myocardial infarction. Acad Emerg Med.

[10] Sun BC, Adams J, Orav E, Rucker DW, Brennan TA, Burstin HR (2000). Determinants of patient satisfaction and willingness to return with emergency care. Annals Emergency Med.

[11] Krochmal P, Riley TA (1994). Increased health care costs associated with ED overcrowding. Am J Emerg Med.

[12] Adriaenssens J, De Gucht V, Maes S (2015). Determinants and prevalence of burnout in emergency nurses: a systematic review of 25 years of research. Int J Nurs Stud.

[13] Kulstad EB, Sikka R, Sweis RT, Kelley KM, Rzechula KH (2010). ED overcrowding is associated with an increased frequency of medication errors. Am J Emerg Med.

[14] Morley C, Unwin M, Peterson GM, Stankovich J, Kinsman L (2018). Emergency department crowding: a systematic review of causes, consequences and solutions. PLoS One.

[15] Kirubarajan A, Taher A, Khan S, Masood S (2020). Artificial intelligence in emergency medicine: a scoping review. J Am Coll Emerg Physicians Open.

[16] Mathews SC, McShea MJ, Hanley CL, Ravitz A, Labrique AB, Cohen AB (2019). Digital health: a path to validation. NPJ Digit Med.

[17] Lewis TL, Wyatt JC (2014). mHealth and mobile medical Apps: a framework to assess risk and promote safer use. J Med Internet Res.

[18] Boulos MN, Brewer AC, Karimkhani C, Buller DB, Dellavalle RP (2014). Mobile medical and health apps: state of the art, concerns, regulatory control and certification. Online J Public Health Inform.

[19] Hill MG, Sim M, Mills B (2020). The quality of diagnosis and triage advice provided by free online symptom checkers and apps in Australia. Med J Aust.

[20] Semigran H, Linder J, Gidengil C, Mehrotra A (2015). Evaluation of symptom checkers for self diagnosis and triage: audit study. BMJ.

[21] Chambers D, Cantrell AJ, Johnson M, Preston L, Baxter SK, Booth A, Turner J (2019). Digital and online symptom checkers and health assessment/triage services for urgent health problems: systematic review. BMJ Open.

[22] Schmieding ML, Kopka M, Schmidt K, Schulz-Niethammer S, Balzer F, Feufel MA (2022). Triage accuracy of symptom checker apps: 5-year follow-up evaluation. J Med Internet Res.

[23] Baker A, Perov Y, Middleton K, Baxter J, Mullarkey D, Sangar D, Butt M, DoRosario A, Johri S (2020). A comparison of artificial intelligence and human doctors for the purpose of triage and diagnosis. Front Artif Intell.

[24] Middleton K, Butt M, Hammerla N, Hamblin S, Mehta K, Parsa A Sorting out symptoms: design and evaluation of the 'babylon check' automated triage system. ArXiv.

[25] Bellika J, Marco L, Wynn R (2015). A communicable disease query engine. Digital Healthcare Empowering Europeans.

[26] Arnold RJ, Layton A (2015). Cost analysis and clinical outcomes of ambulatory care monitoring in medicare patients: describing the diagnostic odyssey. J Health Econ Outcomes Res.

[27] Miller S, Gilbert S, Virani V, Wicks P (2020). Patients' utilization and perception of an artificial intelligence-based symptom assessment and advice technology in a British primary care waiting room: exploratory pilot study. JMIR Hum Factors.

[28] Armstrong S (2018). The apps attempting to transfer NHS 111 online. BMJ.

[29] Babylon Health homepage. Babylon Health.

[30] Ayanouz S, Abdelhakim B, Benhmed M (2020). A smart chatbot architecture based NLP and machine learning for health care assistance. Proceedings of the 3rd International Conference on Networking, Information Systems & Security.

[31] babylonhealth / neuralTPPs. GitHub.

[32] Enguehard J, Busbridge D, Bozson A, Woodcock C, Hammerla NY Neural temporal point processes for modelling electronic health records. ArXiv.

[33] Pittet V, Burnand B, Yersin B, Carron P (2014). Trends of pre-hospital emergency medical services activity over 10 years: a population-based registry analysis. BMC Health Serv Res.

[34] Cabral EL, Castro WR, Florentino DR, Viana DD, Costa Junior JF, Souza RP, Rêgo AC, Araújo-Filho I, Medeiros AC (2018). Response time in the emergency services. Systematic review. Acta Cir Bras.

[35] Lowthian JA, Cameron PA, Stoelwinder JU, Curtis A, Currell A, Cooke MW, McNeil JJ (2011). Increasing utilisation of emergency ambulances. Aust Health Rev.

[36] Byrsell F, Claesson A, Ringh M, Svensson L, Jonsson M, Nordberg P, Forsberg S, Hollenberg J, Nord A (2021). Machine learning can support dispatchers to better and faster recognize out-of-hospital cardiac arrest during emergency calls: a retrospective study. Resuscitation.

[37] Borgholt L, Havtorn J, Agić Ž, Søgaard A, Maaløe L, Igel C (2020). Do end-to-end speech recognition models care about context?. Proceedings of the Interspeech 2020.

[38] Graves A, Fernández S, Gomez F, Schmidhuber J (2006). Connectionist temporal classification: labelling unsegmented sequence data with recurrent neural networks. Proceedings of the 23rd international conference on Machine learning.

[39] Miller M, Bootland D, Jorm L, Gallego B (2022). Improving ambulance dispatch triage to trauma: a scoping review using the framework of development and evaluation of clinical prediction rules. Injury.

[40] Vaswani A, Shazeer N, Parmar N, Uszkoreit J, Jones L, Gomez AN, Kaiser L, Polosukhin I Attention is all you need. arXiv.

[41] Devlin J, Chang M-W, Lee K, Toutanova K BERT: pre-training of deep bidirectional transformers for language understanding. arXiv.

[42] Radford A, Wu J, Child R, Luan D, Amodei D, Sutskever I (2019). Language models are unsupervised multitask learners. OpenAI blog.

[43] Rasmy L, Xiang Y, Xie Z, Tao C, Zhi D (2021). Med-BERT: pretrained contextualized embeddings on large-scale structured electronic health records for disease prediction. NPJ Digit Med.

[44] Naseem U, Dunn AG, Khushi M, Kim J (2022). Benchmarking for biomedical natural language processing tasks with a domain specific ALBERT. BMC Bioinformatics.

[45] Amin-Nejad A, Ive J, Velupillai S (2020). Exploring transformer text generation for medical dataset augmentation. Proceedings of the Twelfth Language Resources and Evaluation Conference.

[46] Gopinath D, Agrawal M, Murray L, Horng S, Karger D, Sontag D Fast, structured clinical documentation via contextual autocomplete. arXiv.

[47] Af Ugglas B, Skyttberg N, Wladis A, Djärv T, Holzmann MJ (2020). Emergency department crowding and hospital transformation during COVID-19, a retrospective, descriptive study of a university hospital in Stockholm, Sweden. Scand J Trauma Resusc Emerg Med.

[48] Saberian P, Conovaloff J, Vahidi E, Hasani-Sharamin P, Kolivand P (2020). How the COVID-19 epidemic affected prehospital emergency medical services in Tehran, Iran. West J Emerg Med.

[49] 911 Overflow. Devpost.

[50] Pfeifer R, Halvachizadeh S, Schick S, Sprengel K, Jensen KO, Teuben M, Mica L, Neuhaus V, Pape H (2019). Are pre-hospital trauma deaths preventable? A systematic literature review. World J Surg.

[51] Tollinton L, Metcalf AM, Velupillai S (2020). Enhancing predictions of patient conveyance using emergency call handler free text notes for unconscious and fainting incidents reported to the London Ambulance Service. Int J Med Inform.

[52] Ferri P, Sáez C, Félix-De Castro A, Juan-Albarracín J, Blanes-Selva V, Sánchez-Cuesta P, García-Gómez JM (2021). Deep ensemble multitask classification of emergency medical call incidents combining multimodal data improves emergency medical dispatch. Artif Intell Med.

[53] Fix J, Ising AI, Proescholdbell SK, Falls DM, Wolff CS, Fernandez AR, Waller AE (2021). Linking emergency medical services and emergency department data to improve overdose surveillance in North Carolina. Public Health Rep.

[54] Martin T, Ranney M, Dorroh J, Asselin N, Sarkar I (2018). Health information exchange in emergency medical services. Appl Clin Inform.

[55] Redfield C, Tlimat A, Halpern Y, Schoenfeld DW, Ullman E, Sontag DA, Nathanson LA, Horng S (2020). Derivation and validation of a machine learning record linkage algorithm between emergency medical services and the emergency department. J Am Med Inform Assoc.

[56] Kirkland SW, Soleimani A, Rowe BH, Newton AS (2019). A systematic review examining the impact of redirecting low-acuity patients seeking emergency department care: is the juice worth the squeeze?. Emerg Med J.

[57] Worster A, Fernandes CM, Eva K, Upadhye S (2007). Predictive validity comparison of two five-level triage acuity scales. Eur J Emergency Med.

[58] Farrohknia N, Castrén M, Ehrenberg A, Lind L, Oredsson S, Jonsson H, Asplund K, Göransson KE (2011). Emergency department triage scales and their components: a systematic review of the scientific evidence. Scand J Trauma Resusc Emerg Med.

[59] Christ M, Grossmann F, Winter D, Bingisser R, Platz E (2010). Modern triage in the emergency department. Dtsch Arztebl Int.

[60] Hinson JS, Martinez DA, Cabral S, George K, Whalen M, Hansoti B, Levin S (2019). Triage performance in emergency medicine: a systematic review. Ann Emerg Med.

[61] Fernandes M, Vieira SM, Leite F, Palos C, Finkelstein S, Sousa JM (2020). Clinical decision support systems for triage in the emergency department using intelligent systems: a review. Artif Intell Med.

[62] Levin S, Toerper M, Hamrock E, Hinson JS, Barnes S, Gardner H, Dugas A, Linton B, Kirsch T, Kelen G (2018). Machine-learning-based electronic triage more accurately differentiates patients with respect to clinical outcomes compared with the emergency severity index. Annal Emergency Med.

[63] Ivanov O, Wolf L, Brecher D, Lewis E, Masek K, Montgomery K, Andrieiev Y, McLaughlin M, Liu S, Dunne R, Klauer K, Reilly C (2021). Improving ED emergency severity index acuity assignment using machine learning and clinical natural language processing. J Emerg Nurs.

[64] Chen T, Guestrin C (2016). XGBoost: a scalable tree boosting system. Proceedings of the 22nd ACM SIGKDD International Conference on Knowledge Discovery and Data Mining.

[65] Sinsky C, Colligan L, Li L, Prgomet M, Reynolds S, Goeders L, Westbrook J, Tutty M, Blike G (2016). Allocation of physician time in ambulatory practice: a time and motion study in 4 specialties. Ann Intern Med.

[66] Gardner R, Cooper E, Haskell J, Harris DA, Poplau S, Kroth PJ, Linzer M (2019). Physician stress and burnout: the impact of health information technology. J Am Med Inform Assoc.

[67] Carayon P, Wetterneck TB, Alyousef B, Brown RL, Cartmill RS, McGuire K, Hoonakker PL, Slagle J, Van Roy KS, Walker JM, Weinger MB, Xie A, Wood KE (2015). Impact of electronic health record technology on the work and workflow of physicians in the intensive care unit. Int J Med Inform.

[68] Chan KS, Fowles JB, Weiner JP (2010). Review: electronic health records and the reliability and validity of quality measures: a review of the literature. Med Care Res Rev.

[69] Kossman SP, Scheidenhelm SL (2008). Nurses' perceptions of the impact of electronic health records on work and patient outcomes. Comput Inform Nurs.

[70] Smith CA, Hetzel S, Dalrymple P, Keselman A (2011). Beyond readability: investigating coherence of clinical text for consumers. J Med Internet Res.

[71] Grigonyte G, Kvist M, Velupillai S, Wirén M (2014). Improving readability of Swedish electronic health records through lexical simplification: first results. Proceedings of the 3rd Workshop on Predicting and Improving Text Readability for Target Reader Populations (PITR).

[72] Greenbaum N, Jernite Y, Halpern Y, Calder S, Nathanson LA, Sontag D, Horng S (2017). Contextual autocomplete: a novel user interface using machine learning to improve ontology usage and structured data capture for presenting problems in the emergency department. bioRxiv.

[73] Murray L, Gopinath D, Agrawal M, Horng S, Sontag D, Karger D (2021). MedKnowts: unified documentation and information retrieval for electronic health records. Proceedings of the 34th Annual ACM Symposium on User Interface Software and Technology.

[74] Li J (2022). Recent advances in end-to-end automatic speech recognition. APSIPA Transact Signal Inform Process.

[75] (2021). Using an AI assistant to reduce documentation burden in family medicine. American Academy of Family Physicians Innovation Labs Report.

[76] Nuance Dragon Medical One homepage. Nuance Dragon Medical One.

[77] Elayan H, Aloqaily M, Guizani M (2021). Digital twin for intelligent context-aware IoT healthcare systems. IEEE Internet Things J.

[78] Chaou C, Chen H, Chang S, Tang P, Pan S, Yen AM, Chiu T (2017). Predicting length of stay among patients discharged from the emergency department-using an accelerated failure time model. PLoS One.

[79] Bacchi S, Tan Y, Oakden-Rayner L, Jannes J, Kleinig T, Koblar S (2022). Machine learning in the prediction of medical inpatient length of stay. Intern Med J.

[80] Hojat M, Louis DZ, Markham FW, Wender R, Rabinowitz C, Gonnella JS (2011). Physicians' empathy and clinical outcomes for diabetic patients. Acad Med.

[81] Kelley JM, Kraft-Todd G, Schapira L, Kossowsky J, Riess H (2014). The influence of the patient-clinician relationship on healthcare outcomes: a systematic review and meta-analysis of randomized controlled trials. PLoS One.

[82] Richter JP, Muhlestein DB (2017). Patient experience and hospital profitability: is there a link?. Health Care Manage Rev.

[83] Pitrou I, Lecourt A, Bailly L, Brousse B, Dauchet L, Ladner J (2009). Waiting time and assessment of patient satisfaction in a large reference emergency department: a prospective cohort study, France. Eur J Emergency Med.

[84] Thompson DA, Yarnold PR, Williams DR, Adams SL (1996). Effects of actual waiting time, perceived waiting time, information delivery, and expressive quality on patient satisfaction in the emergency department. Ann Emerg Med.

[85] Sonis JD, Aaronson EL, Lee RY, Philpotts LL, White BA (2018). Emergency department patient experience: a systematic review of the literature. J Patient Exp.

[86] Crilly J, Chaboyer W, Creedy D (2004). Violence towards emergency department nurses by patients. Accid Emerg Nurs.

[87] Pich J, Hazelton M, Sundin D, Kable A (2011). Patient-related violence at triage: a qualitative descriptive study. Int Emerg Nurs.

[88] Krishel S, Baraff LJ (1993). Effect of emergency department information on patient satisfaction. Ann Emerg Med.

[89] Hassan R, Twynam N, Nah F, Siau K (2016). Patient engagement in the medical facility waiting room using gamified healthcare information delivery. HCI in Business, Government, and Organizations: Information Systems.

[90] Göransson KE, von Rosen A (2010). Patient experience of the triage encounter in a Swedish emergency department. Int Emerg Nurs.

[91] Broida R, Desai S, Easter B (2016). Emergency department crowding: high impact solutions. Emergency Medicine Practice Committee.

[92] Xie C, Zhang J, Morrison AM, Coca-Stefaniak JA (2021). The effects of risk message frames on post-pandemic travel intentions: the moderation of empathy and perceived waiting time. Current Issues Tourism.

[93] Kilaru AS, Meisel ZF, Paciotti B, Ha YP, Smith RJ, Ranard BL, Merchant RM (2016). What do patients say about emergency departments in online reviews? A qualitative study. BMJ Qual Saf.

[94] Gold JI, Belmont KA, Thomas DA (2007). The neurobiology of virtual reality pain attenuation. Cyberpsychol Behav.

[95] Al-Nerabieah Z, Alhalabi M, Owayda A, Alsabek L, Bshara N, Kouchaji C (2020). Effectiveness of using virtual reality eyeglasses in the waiting room on preoperative anxiety: a randomized controlled trial. Perioperative Care Operating Room Manage.

[96] Dandu KV, Carniol ET, Sanghvi S, Baredes S, Eloy JA (2017). A 10-year analysis of head and neck injuries involving nonpowder firearms. Otolaryngol Head Neck Surg.

[97] Josseran L, Fouillet A, Caillère N, Brun-Ney D, Ilef D, Brucker G, Medeiros H, Astagneau P (2010). Assessment of a syndromic surveillance system based on morbidity data: results from the Oscour network during a heat wave. PLoS One.

[98] Gil-Jardiné C, Chenais G, Pradeau C, Tentillier E, Revel P, Combes X, Galinski M, Tellier E, Lagarde E (2022). Surveillance of COVID-19 using a keyword search for symptoms in reports from emergency medical communication centers in Gironde, France: a 15 year retrospective cross-sectional study. Intern Emerg Med.

[99] Gil-Jardiné C, Chenais G, Pradeau C, Tentillier E, Revel P, Combes X, Galinski M, Tellier E, Lagarde E (2021). Trends in reasons for emergency calls during the COVID-19 crisis in the department of Gironde, France using artificial neural network for natural language classification. Scand J Trauma Resusc Emerg Med.

[100] Sahu KS, Majowicz SE, Dubin JA, Morita PP (2021). NextGen public health surveillance and the Internet of Things (IoT). Front Public Health.

[101] (2022). AI Risk Management Framework: Second Draft. National Institute of Standard and Technology.

[102] (2021). Ethics and Governance of Artificial Intelligence for Health WHO Guidance.

[103] Meskó B, Spiegel B (2022). A revised hippocratic oath for the era of digital health. J Med Internet Res.

[104] ISO/IEC TS 5723:2022(en) Trustworthiness — Vocabulary. ISO.

[105] Zhuang S, Hadfield-Menell D (2020). Consequences of misaligned AI. Proceedings of the 34th International Conference on Neural Information Processing Systems.

[106] D'Amour A, Heller K, Moldovan D, Adlam B, Alipanahi B, Beutel A, Chen C, Deaton J, Eisenstein J, Hoffman MD, Hormozdiari F, Houlsby N, Hou S, Jerfel G, Karthikesalingam A, Lucic M, Ma Y, McLean C, Mincu D, Mitani A, Montanari A, Nado Z, Natarajan V, Nielson C, Osborne TF, Raman R, Ramasamy K, Sayres R, Schrouff J, Seneviratne M, Sequeira S, Suresh H, Veitch V, Vladymyrov M, Wang X, Webster K, Yadlowsky S, Yun T, Zhai X, Sculley D (2022). Underspecification presents challenges for credibility in modern machine learning. J Mach Learn Res.

[107] Passi S, Barocas S (2019). Problem formulation and fairness. Proceedings of the Conference on Fairness, Accountability, and Transparency.

[108] Amodei D, Olah C, Steinhardt J, Christiano P, Schulman J, Mané D (2016). Concrete problems in AI safety. arXiv.

[109] Hardt M, Barocas S, Narayanan A (2023). Fairness and Machine Learning Limitations and Opportunities.

[110] Mehrabi N, Morstatter F, Saxena N, Lerman K, Galstyan A (2021). A survey on bias and fairness in machine learning. ACM Comput Surv.

[111] Milner KA, Vaccarino V, Arnold AL, Funk M, Goldberg RJ (2004). Gender and age differences in chief complaints of acute myocardial infarction (Worcester Heart Attack Study). Am J Cardiol.

[112] Bozkurt B, Khalaf S (2017). Heart failure in women. Methodist Debakey Cardiovasc J.

[113] Suresh H, Guttag J (2021). A framework for understanding sources of harm throughout the machine learning life cycle. Proceedings of the Equity and Access in Algorithms, Mechanisms, and Optimization.

[114] Blyth CR (1972). On simpson's paradox and the sure-thing principle. J Am Statistical Assoc.

[115] Forbes L, Canner J, Milio L, Halscott T, Vaught A (2021). Association of patient sex and pregnancy status with naloxone administration during emergency department visits. Obstet Gynecol.

[116] Gehlke CE, Biehl K (1934). Certain effects of grouping upon the size of the correlation coefficient in census tract material. J Am Statistical Assoc.

[117] Kok MR, Tuson M, Yap M, Turlach B, Boruff B, Vickery A, Whyatt D (2021). Impact of the modifiable areal unit problem in assessing determinants of emergency department demand. Emerg Med Australas.

[118] Reimer AP, Milinovich A, Madigan EA (2016). Data quality assessment framework to assess electronic medical record data for use in research. Int J Med Inform.

[119] Taylor RA, Pare JR, Venkatesh AK, Mowafi H, Melnick ER, Fleischman W, Hall MK (2016). Prediction of in-hospital mortality in emergency department patients with sepsis: a local big data-driven, machine learning approach. Acad Emerg Med.

[120] Barberà-Mariné MG, Cannavacciuolo L, Ippolito A, Ponsiglione C, Zollo G (2019). The weight of organizational factors on heuristics. Manage Decision.

[121] Mohan D, Fischhoff B, Angus DC, Rosengart MR, Wallace DJ, Yealy DM, Farris C, Chang CH, Kerti S, Barnato AE (2018). Serious games may improve physician heuristics in trauma triage. Proc Natl Acad Sci U S A.

[122] Hovy D, Prabhumoye S (2021). Five sources of bias in natural language processing. Lang Linguist Compass.

[123] Paullada A, Raji ID, Bender EM, Denton E, Hanna A (2021). Data and its (dis)contents: a survey of dataset development and use in machine learning research. Patterns (N Y).

[124] Schrader CD, Lewis LM (2013). Racial disparity in emergency department triage. J Emerg Med.

[125] Arslanian-Engoren C (2000). Gender and age bias in triage decisions. J Emerg Nurs.

[126] Bender E, Gebru T, McMillan-Major A, Shmitchell S (2021). On the dangers of stochastic parrots: can language models be too big?. Proceedings of the 2021 ACM Conference on Fairness, Accountability, and Transparency.

[127] Wagner C, Garcia D, Jadidi M, Strohmaier M (2021). It’s a man’s wikipedia? Assessing gender inequality in an online encyclopedia. Proc Int AAAI Conf Web Social Media.

[128] Liang P, Li I, Zheng E, Lim Y, Salakhutdinov R, Morency L-P (2020). Towards debiasing sentence representations. Proceedings of the 58th Annual Meeting of the Association for Computational Linguistics.

[129] Liu X, Rivera SC, Moher D, Calvert MJ, Denniston AK, SPIRIT-AICONSORT-AI Working Group (2020). Reporting guidelines for clinical trial reports for interventions involving artificial intelligence: the CONSORT-AI Extension. BMJ.

[130] Rivera SC, Liu X, Chan A, Denniston AK, Calvert MJ, SPIRIT-AICONSORT-AI Working Group (2020). Guidelines for clinical trial protocols for interventions involving artificial intelligence: the SPIRIT-AI Extension. BMJ.

[131] (2018). Intellectual Property and Digital Trade in the Age of Artificial Intelligence and Big Data, Global Perspectives for the Intellectual Property System.

[132] Gerke S, Minssen T, Cohen G (2020). Chapter 12 - Ethical and legal challenges of artificial intelligence-driven healthcare. Artificial Intelligence in Healthcare.

[133] Maliha G, Gerke S, Cohen IG, Parikh RB (2021). Artificial intelligence and liability in medicine: balancing safety and innovation. Milbank Q.

[134] Price WN, Gerke S, Cohen IG (2019). Potential liability for physicians using artificial intelligence. JAMA.

[135] Antoniadi AM, Du Y, Guendouz Y, Wei L, Mazo C, Becker BA, Mooney C (2021). Current challenges and future opportunities for XAI in machine learning-based clinical decision support systems: a systematic review. Applied Sci.

[136] Danilevsky M, Qian K, Aharonov R, Katsis Y, Kawas B, Sen P (2020). A survey of the state of explainable AI for natural language processing. Proceedings of the 1st Conference of the Asia-Pacific Chapter of the Association for Computational Linguistics and the 10th International Joint Conference on Natural Language Processing.

[137] Barredo Arrieta A, Díaz-Rodríguez N, Del Ser J, Bennetot A, Tabik S, Barbado A, Garcia S, Gil-Lopez S, Molina D, Benjamins R, Chatila R, Herrera F (2020). Explainable Artificial Intelligence (XAI): concepts, taxonomies, opportunities and challenges toward responsible AI. Inform Fusion.

[138] Castelvecchi D (2016). Can we open the black box of AI?. Nature.

[139] (1957). Deering's California codes annotated.

[140] Moore G, Matlock A, Kiley J, Percy K (2018). Emergency physicians: beware of the consent standard of care. Clin Pract Cases Emerg Med.

[141] (2022). Proposal for a DIRECTIVE OF THE EUROPEAN PARLIAMENT AND OF THE COUNCIL on adapting non-contractual civil liability rules to artificial intelligence (AI Liability Directive). European Commission.

[142] (2022). New liability rules on products and AI to protect consumers and foster innovation. European Commission.

[143] Forcier M, Gallois H, Mullan S, Joly Y (2019). Integrating artificial intelligence into health care through data access: can the GDPR act as a beacon for policymakers?. J Law Biosci.

